# Supplementary material for: Effect of Germination on Alfalfa and Buckwheat: Phytochemical Profiling by UHPLC-ESI-QTOF-MS/MS, Bioactive Compounds, and In-Vitro Studies of Their Diabetes and Obesity-Related Functions
Source: Antioxidants (Basel). 2021 Oct 13;10(10):1613. doi: 10.3390/antiox10101613 (PMC8533172; doi:10.3390/antiox10101613)
Supplement: Supplementary file 1 [file antioxidants-10-01613-s001.zip › antioxidants-1408852-supplementary.pdf]

**Supplementary Table S1**. The levels of the 33 compounds identified from alfalfa and Buckwheat seed and sprouts extracts by *UHPLC-Q-TOF-MS²* confirmed by comparison with reference standards.

| No. | Metabolites | AL sprout | AL seed | BW sprout | BW seed |
|---|---|---|---|---|---|
| 1. | Lysine | 150000.00 | 0.00 | 28000.00 | 0.00 |
| 2. | L-Arginine | 91000.00 | 77000.00 | 220000.00 | 0.00 |
| 3. | L-Phenylalanine | 850000.00 | 0.00 | 0.00 | 0.00 |
| 4. | Leucine | 220000.00 | 0.00 | 0.00 | 0.00 |
| 5. | L-Valine | 310000.00 | 0.00 | 54000.00 | 0.00 |
| 6. | L-Tryptophan | 200000.00 | 0.00 | 170000.00 | 68000.00 |
| 7. | L-Histidine | 560000.00 | 0.00 | 130000.00 | 0.00 |
| 8. | D-ornithine | 380000.00 | 0.00 | 120000.00 | 0.00 |
| 9. | L-Asparagine | 0.00 | 54000.00 | 54000.00 | 0.00 |
| 10. | γ-Aminobutryic acid | 0.00 | 0.00 | 110000.00 | 0.00 |
| 11. | Pyroglutamic acid | 150000.00 | 48000.00 | 160000.00 | 69000.00 |
| 12. | DL-Homoserine | 240000.00 | 190000.00 | 95000.00 | 0.00 |
| 13. | L-Glutamate | 500000.00 | 0.00 | 200000.00 | 220000.00 |
| 14. | Glutathione | 0.00 | 0.00 | 100000.00 | 0.00 |
| 15. | Uridine | 0.00 | 100000.00 | 55000.00 | 0.00 |
| 16. | Actinonin | 310000.00 | 0.00 | 0.00 | 420000.00 |
| 17. | DL-o-Tyrosine | 250000.00 | 0.00 | 120000.00 | 0.00 |
| 18. | Citric acid | 100000.00 | 770000.00 | 600000.00 | 790000.00 |
| 19. | Malic acid | 64000.00 | 300000.00 | 0.00 | 380000.00 |
| 20. | Lactic acid | 130000.00 | 45000.00 | 0.00 | 0.00 |
| 21. | Fumaric acid | 0.00 | 96000.00 | 0.00 | 120000.00 |
| 22. | 3-Furoic acid | 0.00 | 250000.00 | 190000.00 | 260000.00 |
| 23. | Trans-Cinnamic acid | 140000.00 | 0.00 | 0.00 | 0.00 |
| 24. | m-Coumaric acid | 0.00 | 0.00 | 95000.00 | 0.00 |
| 25. | Ascorbic acid | 66000.00 | 0.00 | 0.00 | 0.00 |
| 26. | (-)-Catechin gallate | 0.00 | 0.00 | 60000.00 | 310000.00 |
| 27. | +)-Epicatechin | 0.00 | 0.00 | 200000.00 | 350000.00 |
| 28. | Gluconic acid | 1600000 | 0.00 | 1700000 | 0.00 |
| 29. | Galactaric acid | 280000 | 0.00 | 0.00 | 0.00 |
| 30. | Hypoxanthine | 260000 | 0.00 | 0.00 | 0.00 |
| 31. | N-Methylglutamic acid | 0.00 | 45000 | 0.00 | 0.00 |
| 32. | 1-Hexadecylamine | 230000 | 490000 | 500000 | 540000 |
| 33. | Adenosine | 0.00 | 560000 | 0.00 | 370000 |

The values are presented as peak area representing the levels of metabolites in the extracts. Zero (0.00) peak areas indicate that the corresponding metabolite was not detected in the respective extract
